# Safety of nivolumab monotherapy in five cancer types: pooled analysis of post-marketing surveillance in Japan

**DOI:** 10.1007/s10147-024-02515-1

**Published:** 2024-06-06

**Authors:** Kenji Hiraizumi, Chikara Honda, Ayu Watanabe, Takafumi Nakao, Shuichi Midorikawa, Hiromi Abe, Nobuki Matsui, Tsunehisa Yamamoto, Takahiko Sakamoto

**Affiliations:** 1 Oncology Medical Affairs, Ono Pharmaceutical Co., Ltd., 1-8-2 Kyutaromachi, Chuo-ku, Osaka, 541-8564 Japan; 2 PV Data Strategy, Pharmacovigilance Department, Ono Pharmaceutical Co., Ltd., 2-1-5 Dosho-machi, Chuo-ku, Osaka, 541-8526 Japan; 3 Safety Management Pharmacovigilance Department, Ono Pharmaceutical Co., Ltd., 2-1-5 Dosho-machi, Chuo-ku, Osaka, 541-8526 Japan; 4 Biometrics and Data Sciences, R&D Department, Bristol-Myers Squibb K.K., Otemachi One Tower, 1-2-1 Otemachi, Chiyoda-ku, Tokyo, 100-0004 Japan; 5 Oncology Medical, Bristol-Myers Squibb K.K., Otemachi One Tower, 1-2-1 Otemachi, Chiyoda-ku, Tokyo, 100-0004 Japan; 6 Patient Safety Japan, Bristol-Myers Squibb K.K., Otemachi One Tower, 1-2-1 Otemachi, Chiyoda-ku, Tokyo, 100-0004 Japan

**Keywords:** Nivolumab, Post-marketing surveillance, Real-world clinical practice, Safety, Treatment-related adverse events

## Abstract

**Background:**

Nivolumab has been approved for treating ≥ 10 cancer types. However, there is limited information on the incidence of rare, but potentially serious, treatment-related adverse events (TRAEs), as well as notable TRAEs in patients with certain medical disorders or older patients in Japan.

**Methods:**

We performed pooled analyses of data from published post-marketing surveillance in Japan of nivolumab monotherapy for patients with malignant melanoma, non-small cell lung cancer, renal cell carcinoma, head and neck cancer, and gastric cancer to determine the frequencies of 20 categories of TRAEs of special interest overall and in patient groups with higher perceived safety risks (history of autoimmune disease, interstitial lung disease, tuberculosis, or hepatitis B/C; patients vaccinated during nivolumab treatment; and older patients [≥ 75 years]).

**Results:**

The overall population comprised 7421 patients treated with nivolumab. TRAEs were reported in 49.1% of patients, with grade ≥ 3 TRAEs in 16.7%. Endocrine disorders (14.4%), hepatobiliary disorders (10.9%), and interstitial lung disease (7.0%) were the three most common categories (any grade). The incidences of rare TRAEs with high risk of becoming serious, which occurred in < 1% of patients, were consistent with those in previous reports. The frequencies of TRAEs were not markedly increased in the specified patient groups relative to the overall population.

**Conclusion:**

To our knowledge, this is the largest study examining the safety of nivolumab-treated patients in real-world clinical practice including rare but potentially serious TRAEs. We found no new signals in the safety of nivolumab among the patient groups relative to the overall population, and no additional safety measures are required in these groups.

*Trial registration* UMIN000048892 (overall analysis), JapicCTI-163272 (melanoma), Japic-163271 (non-small cell lung cancer), JapicCTI-184071 (head and neck cancer), JapicCTI-184070 (gastric cancer), and JapicCTI-184069 (renal cell cancer).

**Supplementary Information:**

The online version contains supplementary material available at 10.1007/s10147-024-02515-1.

**Results of the individual post-marketing surveillance were published in:**

- Tahara M, Kiyota N, Nibu KI, Akamatsu A, Hoshino T, Hayashi R (2021) Real-world safety and effectiveness of nivolumab for recurrent or metastatic head and neck cancer in Japan: a post-marketing surveillance. Int J Clin Oncol 26:1619–1627. 10.1007/s10147-021-01949-1
- Uemura H, Tomita Y, Nonomura N, Yoshizaki K, Nakao T, Shinohara N (2022) Real-world safety and effectiveness of nivolumab for advanced renal cell carcinoma in Japan: a post-marketing surveillance. Int J Clin Oncol 27:1061–1067. 10.1007/s10147-022-02155-3
- Uhara H, Tsuchida T, Kiyohara Y, Akamatsu A, Sakamoto T, Yamazaki N (2022) Safety and effectiveness of nivolumab in Japanese patients with malignant melanoma: Final analysis of a post-marketing surveillance. J Dermatol 49:862–871. 10.1111/1346-8138.16432
- Yamaguchi K, Boku N, Muro K, Yoshida K, Baba H, Tanaka S, Akamatsu A, Sano T (2022) Real-world safety and effectiveness of nivolumab in Japanese patients with unresectable advanced or recurrent gastric/gastroesophageal junction cancer that has progressed after chemotherapy: a postmarketing surveillance study. Gastric Cancer 25:245–253. 10.1007/s10120-021-01244-y (Correction: Gastric Cancer (2022) 25:254. 10.1007/s10120-021-01262-w)
- Yamamoto N, Nakanishi Y, Gemma A, Nakagawa K, Sakamoto T, Akamatsu A, Ohe Y (2021) Real-world safety of nivolumab in patients with non-small-cell lung cancer in Japan: Postmarketing surveillance. Cancer Sci 112:4692–4701. 10.1111/cas.15117

## Introduction

Cancer immunotherapy has revolutionized cancer treatment, and immune checkpoint inhibitors (ICIs) are now the standard therapy for many types of cancers. In clinical practice, it is important to promptly detect potential treatment-related adverse events (TRAEs) and treat them appropriately to maximize the therapeutic potential of ICIs. Information about the incidence and time to onset of TRAEs can be useful for this purpose.

Nivolumab is the first humanized anti-human programmed cell death protein 1 (PD-1) monoclonal antibody, and was approved for malignant melanoma (MM) in July 2014 in Japan [1]. Since then, nivolumab has been approved as monotherapy for more than 10 types of cancer. However, the prior clinical trials of nivolumab in each indication [2–7] were not sufficiently powered to detect rare TRAEs that may become serious or are potentially fatal. Moreover, patients with certain medical disorders and older patients were generally excluded or their enrollment was restricted in the clinical trials. Therefore, the safety information for those patients was insufficient.

In accordance with the risk management plan [8], post-marketing surveillance (PMS) of nivolumab in each tumor type was implemented to collect real-world safety data. The results of published PMS have confirmed the safety profile of nivolumab monotherapy for MM [9], non-small cell lung cancer (NSCLC) [10], renal cell carcinoma (RCC) [11], head and neck cancer (HNC) [12], and gastric cancer (GC) [13]. However, the small numbers of patients with clinically relevant disorders or patient background characteristics hindered meaningful analyses of groups of patients in each PMS.

Therefore, we pooled the safety data from the PMS of nivolumab monotherapy for MM, NSCLC, RCC, HNC, and GC, and performed cross-sectional analyses of its safety. Owing to the large population, we took the opportunity to examine the incidence of rare but potentially serious TRAEs, which were considered TRAEs of special interest (TRAESI). In particular, we assessed the TRAESI of nivolumab in patients with relevant medical disorders (autoimmune disease, interstitial lung disease [ILD], tuberculosis, hepatitis B/C) or specific background characteristics (vaccination history, older age), as they may have an impact on the safety or effectiveness of ICIs [14–19]. Although some studies examined the safety of nivolumab among various age-groups, there are no large cross-sectional analyses of nivolumab in older Japanese patients. Therefore, we examined the safety of nivolumab in patients divided into two age-groups (< 75 and ≥ 75 years). We also investigated the exacerbation/recurrence of autoimmune diseases and tuberculosis during treatment with nivolumab.

## Patients and methods

This report describes a pooled analysis of the PMS of nivolumab monotherapy for MM [9], unresectable, advanced or recurrent NSCLC [10], advanced RCC [11], recurrent or metastatic HNC [12], and unresectable advanced or recurrent gastric/gastroesophageal junction cancer [13]. The design and results of each PMS are described in the original publications.

### PMS design and data collection

Patients scheduled to start treatment with nivolumab at a contracted hospital were to be registered in the PMS, with an observation period of 12 months for MM (patient registration period: Jul 4, 2014 to Feb 28, 2017), NSCLC (Dec 17, 2015 to Mar 31, 2016), or RCC (Aug 26, 2016 to Jan 31, 2017), and 6 months for GC (Nov 1, 2017 to Oct 31, 2018) or HNC (Mar 24, 2017 to Jun 30, 2017). Nivolumab was to be prescribed in accordance with the package insert [20] and proper use guide [21].

The case-report forms (CRFs) in each PMS compiled information on patient demographic/clinical characteristics (including coexisting/historical diseases and vaccination [with type of vaccine] during the PMS), nivolumab treatment (line and number of doses), and safety data (frequency, time to onset, grade, and outcome of TRAEs).

The categories of TRAESI are listed in Table 1. Each category aggregated TRAEs with related preferred terms. For patients with an autoimmune disease or tuberculosis, we analyzed the CRFs regarding the relapse/exacerbation of these diseases, together with the time to onset, grade, and outcome.

**Table 1:** Categories of TRAESI

Categories of TRAESI^a^
• Interstitial lung disease
• Myasthenia gravis
• Myocarditis
• Myositis
• Rhabdomyolysis
• Gastrointestinal disorders (colitis, enteritis, severe diarrhea)
• Type 1 diabetes mellitus
• Hepatobiliary disorders (hepatitis fulminant, hepatic failure, hepatic impairment, hepatitis, cholangitis sclerosing)
• Endocrine disorders (thyroid disorders, pituitary disorders, adrenal disorders)
• Nervous system disorders
• Renal and urinary disorders
• Encephalitis
• Severe skin disorders
• Venous thromboembolism
• Infusion reactions (within 24 h)
• Cardiac disorders
• Serious blood disorders
• Hemophagocytic syndrome
• Pancreatitis
• Tuberculosis

*TRAESI* treatment-related adverse events of special interest

^a^Each category aggregates TRAEs with related preferred terms

### Data analysis

The data were analyzed for the overall (safety analysis) population, which comprised all patients for whom the CRF was completed, excluding those for whom the institution did not provide consent for publication, patients who were not treated with nivolumab, duplicate registrations, and violation of registration, as previously reported [9–13].

## Results

### Patients

**Fig. 1:**
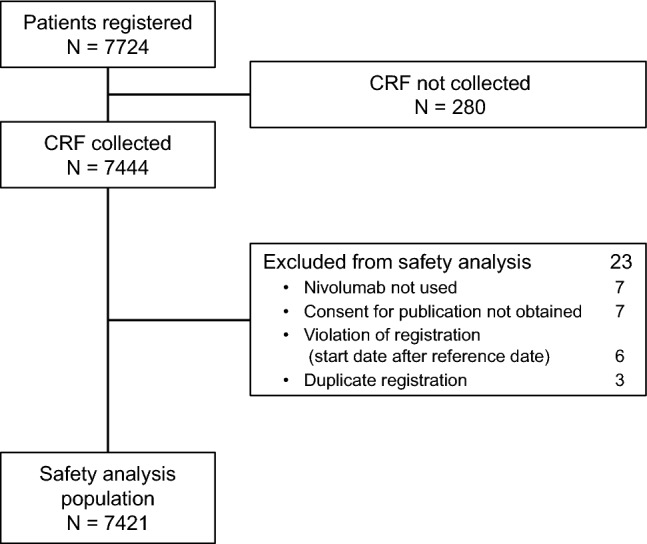
Patient disposition. *CRF* case-report form

A total of 7724 patients were registered and CRFs were completed for 7444 patients. After excluding 23 patients for the reasons listed in Fig. 1, the overall population comprised 7421 patients (safety analysis set). The general characteristics, including the type of cancer and medical histories, of the overall population are summarized in Table 2. Males and females comprised 67.1% and 32.9% of patients, respectively. The median age was 67.0 (range 14–98) years, and 21.8% were ≥ 75 years old. MM, NSCLC, RCC, HNC, and GC accounted for 26.7%, 48.5%, 7.5%, 8.2%, and 8.8% of the patients, respectively. Twenty-five patients received nivolumab for off-label purposes (23 in the MM PMS [9] and 2 in the NSCLC PMS [10]) and were included in the safety analyses here. Overall, 2.9% of patients had a history of autoimmune disease, 3.9% had a history of ILD, 1.1% had a history of tuberculosis, 1.2% had a history of hepatitis B, and 0.9% had a history of hepatitis C. Eighty-nine patients (1.2%) were vaccinated during the PMS, 77 for influenza, and 12 for *Streptococcus pneumoniae*. The distribution of nivolumab doses by indication is shown in ESM Table 1.

**Table 2:** Characteristics of the overall population of patients

Patient characteristics	Value
Overall	7421 (100.0)
Sex	
Male	4980 (67.1)
Female	2441 (32.9)
Age	
< 75 years	5802 (78.2)
≥ 75 years	1619 (21.8)
Median (range), years	67.0 (14–98)
ECOG performance status	
0–1	6078 (81.9)
2–4	1341 (18.1)
Unknown	2 (< 0.1)
Cancer type	
MM	1985 (26.7)
NSCLC	3599 (48.5)
RCC	555 (7.5)
HNC	607 (8.2)
GC	650 (8.8)
Other (off-label use in the PMS)	25 (0.3)
History of autoimmune disease	214 (2.9)
Unknown	7 (< 0.1)
History of ILD	290 (3.9)
History of tuberculosis	84 (1.1)
History of hepatitis B	89 (1.2)
History of hepatitis C	68 (0.9)
Combination with vaccination	
No	7326 (98.7)
Yes	89 (1.2)
Influenza	77 (86.5)^a^
*Streptococcus pneumoniae*	12 (13.5)^a^
Unknown	6 (< 0.1)
Number of previous treatments	
0	725 (9.8)
1	1728 (23.3)
2	1941 (26.2)
≥ 3	2885 (38.9)
Unknown	142 (1.9)

Values are number (%) of patients unless otherwise specified

*ECOG* Eastern Cooperative Oncology Group, *GC* gastric cancer, *HNC* head and neck cancer, *ILD* interstitial lung disease, *MM* malignant melanoma, *NSCLC* non-small cell lung cancer, *PMS* post-marketing surveillance, *RCC* renal cell carcinoma

^a^The denominator was 89 patients with vaccination

### TRAEs in the overall population

TRAEs were reported in 49.1% of patients, with grade ≥ 3 TRAEs in 16.7% (ESM Table 2). This table also shows the frequencies of TRAEs by system organ class (SOC) in the overall population.

The frequencies of TRAESI (any grade and grade ≥ 3) are listed in Table 3. Endocrine disorders (14.4%), hepatobiliary disorders (10.9%), ILD (7.0%), gastrointestinal disorders (5.5%), and infusion reaction within 24 h (5.3%) were the five most common categories of TRAESI (any grade). The five most common grade ≥ 3 TRAESI were hepatobiliary disorders (3.1%), ILD (2.9%), gastrointestinal disorders (1.5%), endocrine disorders (1.4%), and type 1 diabetes mellitus (T1DM) (0.5%). Rare TRAESI that have a high risk of becoming serious (e.g. myasthenia gravis, myocarditis, rhabdomyolysis, T1DM, encephalitis, venous thromboembolism) are also listed in Table 3. These TRAE categories occurred in < 1% of patients, but most of the events were grade ≥ 3, including life-threatening and fatal events.

**Table 3:** TRAESI in the overall population (N = 7421)

Category^a^	Any grade	Grade ≥ 3			
		All	Grade 3	Grade 4	Grade 5
Interstitial lung disease	523 (7.0)	212 (2.9)	129 (1.7)	32 (0.4)	51 (0.7)
Myasthenia gravis	12 (0.2)	8 (0.1)	4 (< 0.1)	3 (< 0.1)	1 (< 0.1)
Myocarditis	4 (< 0.1)	3 (< 0.1)	1 (< 0.1)	1 (< 0.1)	1 (< 0.1)
Myositis	22 (0.3)	12 (0.2)	9 (0.1)	2 (< 0.1)	1 (< 0.1)
Rhabdomyolysis	5 (< 0.1)	4 (< 0.1)	2 (< 0.1)	1 (< 0.1)	1 (< 0.1)
Gastrointestinal disorders	405 (5.5)	114 (1.5)	93 (1.3)	13 (0.2)	8 (0.1)
Type 1 diabetes mellitus	37 (0.5)	34 (0.5)	12 (0.2)	21 (0.3)	1 (< 0.1)
Hepatobiliary disorders	811 (10.9)	227 (3.1)	166 (2.2)	46 (0.6)	15 (0.2)
Endocrine disorders	1070 (14.4)	103 (1.4)	83 (1.1)	14 (0.2)	6 (< 0.1)
Nervous system disorders	44 (0.6)	9 (0.1)	9 (0.1)	0	0
Renal and urinary disorders	110 (1.5)	27 (0.4)	17 (0.2)	4 (< 0.1)	6 (< 0.1)
Encephalitis	4 (< 0.1)	3 (< 0.1)	2 (< 0.1)	0	1 (< 0.1)
Severe skin disorders	45 (0.6)	29 (0.4)	23 (0.3)	6 (< 0.1)	0
Venous thromboembolism	32 (0.4)	18 (0.2)	10 (0.1)	3 (< 0.1)	5 (< 0.1)
Infusion reaction (within 24 h)	393 (5.3)	22 (0.3)	17 (0.2)	2 (< 0.1)	3 (< 0.1)
Cardiac disorders	62 (0.8)	28 (0.4)	12 (0.2)	5 (< 0.1)	11 (0.1)
Serious blood disorders	14 (0.2)	13 (0.2)	3 (< 0.1)	8 (0.1)	2 (< 0.1)
Hemophagocytic syndrome	5 (< 0.1)	4 (< 0.1)	2 (< 0.1)	2 (< 0.1)	0
Pancreatitis	4 (< 0.1)	1 (< 0.1)	1 (< 0.1)	0	0
Tuberculosis	2 (< 0.1)	0	0	0	0

Values are number (%) of patients

*TRAESI* treatment-related adverse events of special interest

^a^Each category aggregates TRAEs with related preferred terms

The median time to the onset of TRAESI was mostly within 90 days (ESM Fig. 1). However, the median time to onset exceeded 90 days for severe skin disorders (median [range]: 93.5 [4–350] days), hemophagocytic syndrome (94.5 [28–258] days), serious blood disorders (119.0 [4–323] days), T1DM (146.0 [13–341] days), and tuberculosis (173.0 [148–198] days).

The five most common TRAEs were hypothyroidism (8.1%), ILD (5.0%), aspartate aminotransferase increased (4.3%), diarrhea (4.3%), and alanine aminotransferase increased (3.5%). The five most common grade ≥ 3 TRAEs were ILD (2.1%), hepatic function abnormal (1.0%), diarrhea (0.8%), aspartate aminotransferase increased (0.7%), and alanine aminotransferase increased (0.6%).

### TRAEs in prespecified subgroups of patients

#### Autoimmune disease

TRAEs occurred in 61.2% of patients with a medical history of autoimmune disease, with grade ≥ 3 TRAEs in 20.6% (ESM Table 3). This table also shows the frequencies of TRAE SOCs in these patients.

The TRAESI are listed in Table 4; the three most common were endocrine disorders (19.6%), hepatobiliary disorders (15.0%), and ILD (12.6%). ILD (5.1%) and hepatobiliary disorders (3.7%) were the two most common grade ≥ 3 TRAESI.

**Table 4:** TRAESI in patients with a history of autoimmune disease, ILD, tuberculosis, hepatitis B, or hepatitis C

Category^a^	History of autoimmune disease (N = 214)		History of ILD (N = 290)		History of tuberculosis (N = 84)		History of hepatitis B (N = 89)		History of hepatitis C (N = 68)	
	Any grade	Grade ≥ 3	Any grade	Grade ≥ 3	Any grade	Grade ≥ 3	Any grade	Grade ≥ 3	Any grade	Grade ≥ 3
Interstitial lung disease	27 (12.6)	11 (5.1)	70 (24.1)	32 (11.0)	5 (6.0)	3 (3.6)	7 (7.9)	1 (1.1)	4 (5.9)	1 (1.5)
Myasthenia gravis	2 (0.9)	1 (0.5)	0	0	1 (1.2)	1 (1.2)	1 (1.1)	1 (1.1)	0	0
Myocarditis	0	0	0	0	0	0	0	0	0	0
Myositis	1 (0.5)	1 (0.5)	1 (0.3)	1 (0.3)	0	0	0	0	0	0
Rhabdomyolysis	1 (0.5)	0	0	0	0	0	0	0	0	0
Gastrointestinal disorders	8 (3.7)	1 (0.5)	17 (5.9)	4 (1.4)	9 (10.7)	3 (3.6)	1 (1.1)	1 (1.1)	1 (1.5)	0
Type 1 diabetes mellitus	2 (0.9)	2 (0.9)	0	0	0	0	0	0	0	0
Hepatobiliary disorders	32 (15.0)	8 (3.7)	35 (12.1)	11 (3.8)	7 (8.3)	1 (1.2)	15 (16.9)	5 (5.6)	7 (10.3)	2 (2.9)
Endocrine disorders	42 (19.6)	2 (0.9)	44 (15.2)	10 (3.4)	12 (14.3)	0	8 (9.0)	1 (1.1)	6 (8.8)	0
Nervous system disorders	0	0	0	0	0	0	1 (1.1)	1 (1.1)	0	0
Renal and urinary disorders	2 (0.9)	1 (0.5)	8 (2.8)	2 (0.7)	0	0	3 (3.4)	1 (1.1)	0	0
Encephalitis	0	0	1 (0.3)	0	0	0	0	0	0	0
Severe skin disorders	0	0	2 (0.7)	0	1 (1.2)	0	1 (1.1)	1 (1.1)	1 (1.5)	1 (1.5)
Venous thromboembolism	2 (0.9)	0	2 (0.7)	1 (0.3)	0	0	0	0	0	0
Infusion reaction (within 24 h)	13 (6.1)	0	13 (4.5)	0	6 (7.1)	0	6 (6.7)	0	6 (8.8)	0
Cardiac disorders	1 (0.5)	0	3 (1.0)	2 (0.7)	0	0	0	0	1 (1.5)	0
Serious blood disorder	3 (1.4)	2 (0.9)	2 (0.7)	2 (0.7)	0	0	0	0	0	0
Hemophagocytic syndrome	0	0	0	0	0	0	0	0	0	0
Pancreatitis	0	0	0	0	0	0	0	0	0	0
Tuberculosis	0	0	0	0	0	0	0	0	0	0

Values are number (%) of patients

*ILD* interstitial lung disease, *TRAESI* treatment-related adverse events of special interest

^a^Each category aggregates TRAEs with related preferred terms

Exacerbation/recurrence of autoimmune diseases is listed in Table 5. Overall, 16 patients (7.5%) experienced exacerbation/recurrence of their autoimmune disease, which was classified as resolved/resolving in 11 (68.8%). ESM Table 4 shows the frequencies of TRAEs (any) and TRAESI according to the type of preexisting autoimmune disease. The frequencies of TRAEs varied among the types of autoimmune disease.

**Table 5:** Exacerbation/recurrence of autoimmune diseases

Preexisting autoimmune disease	Patients	Exacerbation/recurrence		Recovered/remission^a^	
	N	n (%)		n (%)	
Autoimmune disease	214	16 (7.5)		11 (68.8)	
Type of autoimmune disease					
Rheumatoid arthritis	41	6 (14.6)		4 (66.7)	
Psoriasis	17	5 (29.4)		4 (80.0)	
Myasthenia gravis	5	2 (40.0)		2 (100.0)	
Autoimmune hepatitis	4	1 (25.0)		0	
Rheumatic disorders	4	1 (25.0)		0	
Dermatomyositis	2	1 (50.0)		1 (100.0)	

Values are number (%) of patients

^a^The denominator is the number of patients with exacerbation/recurrence

#### History of ILD

Of 290 patients with a history of ILD, 64.5% experienced any TRAE and 24.5% experienced a grade ≥ 3 TRAE (ESM Table 3). The table also shows the frequency of TRAE SOCs in this patient population. As shown in Table 4, the most common TRAESI were ILD (24.1%), endocrine disorders (15.2%), and hepatobiliary disorders (12.1%). The most common grade ≥ 3 TRAESI were ILD (11.0%), hepatobiliary disorders (3.8%), and endocrine disorders (3.4%). The proportions of patients with resolved/remission ILD, by grade, were similar between those with history of ILD and those without history of ILD (ESM Table 5).

#### History of tuberculosis

Overall, 56.0% of patients with a medical history of tuberculosis experienced any TRAE and 19.0% experienced a grade ≥ 3 TRAE (ESM Table 3). The table also shows the frequency of TRAE SOCs in this patient population.

As shown in Table 4, the most common TRAESI were endocrine disorders (14.3%), gastrointestinal disorders (10.7%), and hepatobiliary disorders (8.3%). Grade ≥ 3 TRAESI were ILD (3.6%), gastrointestinal disorders (3.6%), myasthenia gravis (1.2%), and hepatobiliary disorders (1.2%). No exacerbation/recurrence of tuberculosis was reported. Tuberculosis was reported as a TRAE in 2 patients (< 0.1%) without a medical history of tuberculosis. Both of these TRAEs were classified as grade ≤ 2.

#### Hepatitis B/C

TRAEs occurred in 55.1% of patients with a medical history of hepatitis B and in 45.6% of patients with a medical history of hepatitis C (ESM Table 3). Grade ≥ 3 TRAEs occurred in 23.6% and 14.7% of patients, respectively. This table also shows the frequencies of TRAE SOCs in patients with hepatitis B or C.

In patients with a medical history of hepatitis B, the three most common TRAESI were hepatobiliary disorders (16.9%), endocrine disorders (9.0%), and ILD (7.9%) (Table 4). The most common grade ≥ 3 TRAESI were hepatobiliary disorders (5.6%). In patients with a medical history of hepatitis C, the three most common TRAESI were hepatobiliary disorders (10.3%), endocrine disorders (8.8%), and infusion reaction within 24 h (8.8%). Grade ≥ 3 TRAESI were hepatobiliary disorders (2.9%), ILD (1.5%), and severe skin disorders (1.5%) (Table 4).

#### Patients vaccinated during the PMS

Among patients vaccinated during the PMS, 62.9% experienced any TRAE and 11.2% experienced a grade ≥ 3 TRAE (ESM Table 6). The TRAE SOCs in this patient population are shown in this table.

As shown in Table 6, the three most common TRAESI were endocrine disorders (19.1%), hepatobiliary disorders (13.5%), and gastrointestinal disorders (7.9%). Myositis, gastrointestinal disorders, hepatobiliary disorders, T1DM, renal and urinary disorders, and severe skin disorders were reported as grade ≥ 3 TRAEs in 1 patient (1.1%) each (Table 6).

**Table 6:** TRAESI in patients with a history of vaccination (N = 89)

Category^a^	Any grade	Grade ≥ 3
Interstitial lung disease	4 (4.5)	0
Myasthenia gravis	0	0
Myocarditis	0	0
Myositis	2 (2.2)	1 (1.1)
Rhabdomyolysis	0	0
Gastrointestinal disorders	7 (7.9)	1 (1.1)
Type 1 diabetes mellitus	1 (1.1)	1 (1.1)
Hepatobiliary disorders	12 (13.5)	1 (1.1)
Endocrine disorders	17 (19.1)	0
Nervous system disorders	1 (1.1)	0
Renal and urinary disorders	2 (2.2)	1 (1.1)
Encephalitis	0	0
Severe skin disorders	1 (1.1)	1 (1.1)
Venous thromboembolism	0	0
Infusion reaction (within 24 h)	6 (6.7)	0
Cardiac disorders	1 (1.1)	0
Serious blood disorder	0	0
Hemophagocytic syndrome	0	0
Pancreatitis	0	0
Tuberculosis	0	0

Values are number (%) of patients

*TRAESI* treatment-related adverse events of special interest

^a^Each category aggregates TRAEs with related preferred terms

#### By age: < 75 and ≥ 75 years old

Among patients aged < 75 years, 48.4% experienced any grade TRAEs and 16.5% experienced grade ≥ 3 TRAEs. Among patients aged ≥ 75 years, 51.7% experienced any grade TRAEs and 17.1% experienced grade ≥ 3 TRAEs (ESM Table 7). This table also shows the TRAE SOCs in both age-groups.

Table 7 shows the TRAESI in both age-groups. The three most common TRAESI were the same in patients aged < 75 and those aged ≥ 75 years, comprising endocrine disorders (14.1% and 15.5%, respectively), hepatobiliary disorders (10.9% and 11.1%, respectively), and ILD (6.9% and 7.5%, respectively). The three most frequent grade ≥ 3 TRAESI among patients aged < 75 years were hepatobiliary disorders (3.3%), ILD (2.7%), and gastrointestinal disorders (1.6%). The three most common grade ≥ 3 TRAESI in patients aged ≥ 75 years were ILD (3.3%), hepatobiliary disorders (2.3%), and endocrine disorders (1.5%).

**Table 7:** TRAESI in patients aged < 75 or ≥ 75 years

Category^a^	Patients aged < 75 years (N = 5802)		Patients aged ≥ 75 years (N = 1619)	
	Any grade	Grade ≥ 3	Any grade	Grade ≥ 3
Interstitial lung disease	401 (6.9)	159 (2.7)	122 (7.5)	53 (3.3)
Myasthenia gravis	7 (0.1)	5 (< 0.1)	5 (0.3)	3 (0.2)
Myocarditis	2 (< 0.1)	2 (< 0.1)	2 (0.1)	1 (< 0.1)
Myositis	11 (0.2)	6 (0.1)	11 (0.7)	6 (0.4)
Rhabdomyolysis	2 (< 0.1)	2 (< 0.1)	3 (0.2)	2 (0.1)
Gastrointestinal disorders	327 (5.6)	94 (1.6)	78 (4.8)	20 (1.2)
Type 1 diabetes mellitus	32 (0.6)	29 (0.5)	5 (0.3)	5 (0.3)
Hepatobiliary disorders	632 (10.9)	190 (3.3)	179 (11.1)	37 (2.3)
Endocrine disorders	819 (14.1)	79 (1.4)	251 (15.5)	24 (1.5)
Nervous system disorders	33 (0.6)	6 (0.1)	11 (0.7)	3 (0.2)
Renal and urinary disorders	90 (1.6)	20 (0.3)	20 (1.2)	7 (0.4)
Encephalitis	3 (< 0.1)	2 (< 0.1)	1 (< 0.1)	1 (< 0.1)
Severe skin disorders	33 (0.6)	22 (0.4)	12 (0.7)	7 (0.4)
Venous thromboembolism	26 (0.4)	14 (0.2)	6 (0.4)	4 (0.2)
Infusion reaction (within 24 h)	318 (5.5)	17 (0.3)	75 (4.6)	5 (0.3)
Cardiac disorders	44 (0.8)	18 (0.3)	18 (1.1)	10 (0.6)
Serious blood disorder	9 (0.2)	8 (0.1)	5 (0.3)	5 (0.3)
Hemophagocytic syndrome	4 (< 0.1)	3 (< 0.1)	1 (< 0.1)	1 (< 0.1)
Pancreatitis	2 (< 0.1)	0	2 (0.1)	1 (< 0.1)
Tuberculosis	2 (< 0.1)	0	0	0

Values are number (%) of patients

*TRAESI* treatment-related adverse events of special interest

^a^Each category aggregates TRAEs with related preferred terms

## Discussion

This analysis clarified the safety information of 7421 Japanese patients integrating five previously reported PMS [9–13]. The incidences of TRAESI were similar to the values described in the previous reports. We also report data for rare TRAEs that have a high risk of becoming serious, such as myasthenia gravis, myocarditis, and T1DM. The incidences of these TRAEs are largely consistent with those in previous reports [22–25]. We believe that our report presents valuable data regarding the safety of nivolumab in a Japanese population that are derived from comprehensive analyses of a relatively large dataset comprising multiple cancers. We should, however, consider that the incidences of some TRAEs, including T1DM, may be related to genetic predisposition that differs among ethnicities [26]. Therefore, detailed analysis of the pathogenic factors in the Japanese population, including genetic background, will be important in the future. This pooled analysis was also performed to obtain more insight into the safety of nivolumab in certain groups of patients, namely patients with autoimmune diseases, ILD, tuberculosis, hepatitis B or C, history of vaccination, and older patients (≥ 75 years old).

### Patients with a history of autoimmune disease

In patients with a history of autoimmune disease, the overall frequency of TRAEs (61.2%) and the frequencies of some TRAESI, such as endocrine disorders, hepatobiliary disorders, and ILD, were slightly high relative to those in the overall population. The reason for this is unclear after examining the patient background characteristics and concomitant TRAEs. In the NSCLC PMS [10], autoimmune disease (previous or comorbid disease) was not identified as a risk factor although it was statistically significant in univariate analysis. To our knowledge, there are no previous reports describing a high incidence of specific TRAEs.

Due to the mechanism of action of ICIs, there is a risk of exacerbation or recurrence of underlying autoimmune diseases. In previous reports, exacerbation of a preexisting autoimmune disease occurred in 24–47% of patients treated with an anti-PD-1 antibody [27–29]. Other studies have also suggested that the risk of exacerbation during treatment with an ICI was greater in patients with psoriasis or rheumatoid arthritis [30–37]. However, we should remember that the observation period differed between each study.

In this pooled analysis, exacerbation/recurrence of autoimmune disease was reported in 7.5% of patients. Most of these events were classified as recovered/recovering. Thus, most cases of disease exacerbation or recurrence could be managed appropriately with timely diagnosis of immune-related adverse events (irAEs). However, because patients with autoimmune disease may be prone to autoimmune activation, they should be informed about the risks and benefits of treatment with nivolumab.

### Patients with a history of ILD

In patients with a history of ILD, the incidence of any grade and serious ILD, as a TRAESI, was higher than that of the overall population. The high incidence of TRAEs in these patients was attributed to pulmonary-associated disorders (ESM Table 3). These findings are consistent with previous reports [38, 39], and history of ILD was identified as a risk factor for the development of ILD during nivolumab treatment in the NSCLC PMS [10]. Therefore, these findings are reasonable. We also found that the proportions of patients with recovered/remission ILD were comparable between patients with or without history of ILD (ESM Table 5). Therefore, healthcare professionals should pay attention to ILD when administering nivolumab to patients with a history of ILD.

### Patients with a history of tuberculosis

Because ICIs target T cells, which are important to maintaining immunity against tuberculosis, they may increase the risks of tuberculosis reactivation or de novo infection [40]. Therefore, it is important to evaluate the safety of ICIs in patients with a history of or current tuberculosis infection.

In this pooled analysis, the incidence of gastrointestinal disorders was slightly higher in patients with a history of tuberculosis compared with the overall population. However, the reason for this is unclear after examining the patient background characteristics and concomitant TRAEs. To our knowledge, there are no prior reports showing similar findings. Thus, this difference might be incidental.

No cases of exacerbation/recurrence of tuberculosis were reported, although two patients experienced de novo tuberculosis as a TRAE. Reactivation/relapse and new infections were rare in prior studies [18, 41–46]. Although the risks of exacerbation/relapse or new infection were low, healthcare professionals should be vigilant for possible episodes of tuberculosis.

### Patients with a history of hepatitis B/C

In previous reports, there was no difference in the frequency of ICI-induced liver injury in patients with history of hepatitis B or C [15, 16]. Our results also showed no difference in the frequency of ICI-induced liver injury among patients with history of hepatitis C compared with that of the overall population. However, the frequency of hepatobiliary disorders was slightly higher among patients with a history of hepatitis B compared with the overall population. Lin et al. reported that hepatitis B surface antigen (HBsAg) positivity and hepatitis B virus DNA levels were risk factors for hepatotoxicity [47] and ICIs can reactivate HBV [48], although the reasons were unknown. Considering these findings, healthcare professionals should be vigilant for possible liver-related events in patients with history of hepatitis B.

### Patients with a history of vaccination

The frequency of TRAEs of any grade, but not grade ≥ 3 TRAEs, was slightly higher in patients with a history of vaccination, with slightly higher frequencies of endocrine disorders and hepatobiliary disorders in particular. The reason for this is unclear. One prior study reported a higher incidence of irAEs in patients with a history of vaccination [19] whereas another found no difference [49]. Thus, the impact of vaccination on the safety of ICIs remains unclear. Because vaccination causes immune activation, it is important to be vigilant for possible irAEs in this setting.

### Patients aged ≥ 75 years

To our knowledge, this is the largest study to examine the safety of nivolumab in patients aged ≥ 75 years. Of note, we found no marked differences in the safety of nivolumab between patients aged < 75 and ≥ 75 years, similar to the results of earlier studies that used differing age-groups [50–53]. For example, in a retrospective study of patients treated with an ICI in Japan, Sakakida et al. found no significant differences in the frequencies of irAEs, hospitalization, and treatment discontinuation due to irAEs between patients aged ≥ 75 years and those aged < 75 years [50], similar to our findings. Furthermore, Samani et al. [52] reported that fewer patients aged 65–74 or ≥ 75 years discontinued ICI treatment due to toxicities than patients aged < 65 years. However, Singh et al. [53] reported numerically higher frequencies of AEs (regardless of causality) for nivolumab, and Saleh et al. [51] reported numerically higher frequencies of TRAEs for ICIs in older patients. Thus, physicians should continue to be vigilant for TRAEs in older patients.

### Limitations

The limitations of the PMS are described in the original reports [9–13], and include under-reporting or misclassification of the TRAEs. Furthermore, the results may not be generalizable to clinical practice in other countries. Some bias might also be introduced due to the different numbers of patients with each type of cancer, for which there were differences in the observation periods and nivolumab doses. Therefore, the results reported here may not accurately reflect the actual incidence of TRAEs in the total population of patients treated with nivolumab.

## Conclusions

Here, we have summarized the post-marketing safety, including rare but potentially serious TRAEs, of nivolumab in a pooled analysis of 7421 patients treated in real-world clinical practice in Japan. We focused on certain groups of patients who were under-represented or excluded from prior clinical trials (autoimmune disease, ILD, tuberculosis, hepatitis B or C, vaccination, and older patients). We found no new signals in the safety of nivolumab among these groups of patients and the overall population. Although continuous vigilance is important for the timely detection and management of irAEs/TRAEs, no additional safety measures are required in these patients.

### Supplementary Information

Below is the link to the electronic supplementary material.

Supplementary file 1 (DOCX 106 KB)

## Data Availability

The datasets analyzed during the current study are not publicly available because patient consent for individual data disclosure has not been obtained, but are available from the corresponding author on reasonable request.
