# Supplementary material for: Safety of nivolumab monotherapy in five cancer types: pooled analysis of post-marketing surveillance in Japan
Source: Int J Clin Oncol. 2024 Jun 6;29(7):932–43. doi: 10.1007/s10147-024-02515-1 (PMC11196337; doi:10.1007/s10147-024-02515-1)
Supplement: Supplementary file 1 — (DOCX 106 KB) [file 10147_2024_2515_MOESM1_ESM.docx]

# Electronic Supplementary Material

**Safety of nivolumab monotherapy in five cancer types: pooled analysis of post-marketing surveillance in Japan**

Kenji Hiraizumi ^1^, Chikara Honda ^2^, Ayu Watanabe ^3^, Takafumi Nakao ^3^, Shuichi Midorikawa ^4^, Hiromi Abe ^5^, Nobuki Matsui ^6^, Tsunehisa Yamamoto ^5^, Takahiko Sakamoto ^3^

^1^ Oncology Medical Affairs, Ono Pharmaceutical Co., Ltd., 1-8-2 Kyutaromachi, Chuo‑ku, Osaka, 541‑8564, Japan

^2^ PV Data Strategy, Pharmacovigilance Department, Ono Pharmaceutical Co., Ltd., 2‑1‑5 Dosho‑machi, Chuo‑ku, Osaka, 541‑8526, Japan

^3^ Safety Management Pharmacovigilance Department, Ono Pharmaceutical Co., Ltd., 2‑1‑5 Dosho‑machi, Chuo‑ku, Osaka, 541‑8526, Japan

^4^ Biometrics and Data Sciences, R&D Department, Bristol Myers Squibb K.K., Otemachi, One Tower, 1-2-1 Otemachi Chiyoda-ku, Tokyo, 100-0004, Japan

^5^ Oncology Medical, Bristol Myers Squibb K.K., Otemachi One Tower, 1-2-1 Otemachi, Chiyoda-ku, Tokyo, 100-0004, Japan

^6^ Patient Safety Japan, Bristol Myers Squibb K.K., Otemachi One Tower, 1-2-1 Otemachi, Chiyoda-ku, Tokyo, 100-0004, Japan

**Contents**

|  | **Page** |
| --- | --- |
| **ESM Table 1.** Distribution of nivolumab dosing according to indication/observation period | 2 |
| **ESM Table 2.** TRAE SOCs in the overall population (N = 7421) | 3 |
| **ESM Table 3.** TRAE SOCs in patients with a history of autoimmune diseases, ILD, tuberculosis, hepatitis B, or hepatitis C | 4 |
| **ESM Table 4.** Frequency of TRAEs in patients with preexisting autoimmune diseases | 6 |
| **ESM Table 5.** Incidence and recovery/remission of ILD in patients with or without a history of ILD | 8 |
| **ESM Table 6.** TRAE SOCs in patients with a history of vaccination (N = 89) | 9 |
| **ESM Table 7.** TRAE SOCs in patients aged <75 or ≥75 years | 10 |
| **ESM Figure 1.** Time to onset of TRAESI | 11 |

**ESM Table 1.** Distribution of nivolumab dosing according to indication/observation period

|  | **GC, HNC (N = 1257)** | | **NSCLC, MM, RCC (N = 6164)** | |
| --- | --- | --- | --- | --- |
| Planned observation period | 6 months | | 12 months | |
| Number of doses, n (%) |  | |  | |
| 1–4 | 549 | (43.7) | 2764 | (44.8) |
| 5–8 | 326 | (25.9) | 1110 | (18.0) |
| 9–12 | 153 | (12.2) | 665 | (10.8) |
| 13–16 | 228 | (18.1) | 461 | (7.5) |
| 17–20 | 1 | (<0.1) | 430 | (7.0) |
| 21–24 | 0 |  | 302 | (4.9) |
| ≥25 | 0 |  | 432 | (7.0) |
| Median (range) | 5.0 (1–18) | | 6.0 (1–46) | |

*GC* gastric cancer, *HNC* head and neck cancer, *MM* malignant melanoma, *NSCLC* non-small cell lung cancer, *RCC* renal cell carcinoma

**ESM Table 2.** TRAE SOCs in the overall population (N = 7421)

| **SOC** | **Any grade** | | **Grade ≥3** | |
| --- | --- | --- | --- | --- |
| Any TRAEs | 3643 | (49.1) | 1237 | (16.7) |
| Infections and infestations | 261 | (3.5) | 129 | (1.7) |
| Neoplasms benign, malignant, and unspecified (including cysts and polyps) | 37 | (0.5) | 18 | (0.2) |
| Blood and lymphatic system disorders | 110 | (1.5) | 65 | (0.9) |
| Immune system disorders | 8 | (0.1) | 5 | (<0.1) |
| Endocrine disorders | 914 | (12.3) | 101 | (1.4) |
| Metabolism and nutrition disorders | 300 | (4.0) | 122 | (1.6) |
| Psychiatric disorders | 22 | (0.3) | 7 | (<0.1) |
| Nervous system disorders | 219 | (3.0) | 57 | (0.8) |
| Eye disorders | 60 | (0.8) | 10 | (0.1) |
| Ear and labyrinth disorders | 8 | (0.1) | 0 |  |
| Cardiac disorders | 62 | (0.8) | 28 | (0.4) |
| Vascular disorders | 62 | (0.8) | 24 | (0.3) |
| Respiratory, thoracic and mediastinal disorders | 643 | (8.7) | 268 | (3.6) |
| Gastrointestinal disorders | 621 | (8.4) | 165 | (2.2) |
| Hepatobiliary disorders | 327 | (4.4) | 123 | (1.7) |
| Skin and subcutaneous tissue disorders | 767 | (10.3) | 87 | (1.2) |
| Musculoskeletal and connective tissue disorders | 173 | (2.3) | 37 | (0.5) |
| Renal and urinary disorders | 117 | (1.6) | 35 | (0.5) |
| Reproductive and breast disorders | 6 | (<0.1) | 0 |  |
| Congenital, familial and genetic disorders | 1 | (<0.1) | 1 | (<0.1) |
| General and systemic disorders and administration site conditions | 556 | (7.5) | 54 | (0.7) |
| Investigations | 850 | (11.5) | 188 | (2.5) |
| Injury, poisoning and procedural complications | 221 | (3.0) | 26 | (0.4) |

Values are number (%) of patients

*SOC* system organ class, *TRAE* treatment-related adverse event

**ESM Table 3.** TRAE SOCs in patients with a history of autoimmune diseases, ILD, tuberculosis, hepatitis B, or hepatitis C

| **SOC** | **History of autoimmune disease**  **(N = 214)** | | | | **History of ILD**  **(N = 290)** | | | | **History of tuberculosis**  **(N = 84)** | | | | **History of hepatitis B**  **(N = 89)** | | | | **History of hepatitis C**  **(N = 68)** | | | |
| --- | --- | --- | --- | --- | --- | --- | --- | --- | --- | --- | --- | --- | --- | --- | --- | --- | --- | --- | --- | --- |
|  | **Any grade** | | **Grade ≥3** | | **Any grade** | | **Grade ≥3** | | **Any grade** | | **Grade ≥3** | | **Any grade** | | **Grade ≥3** | | **Any grade** | | **Grade ≥3** | |
| Any TRAEs | 131 | (61.2) | 44 | (20.6) | 187 | (64.5) | 71 | (24.5) | 47 | (56.0) | 16 | (19.0) | 49 | (55.1) | 21 | (23.6) | 31 | (45.6) | 10 | (14.7) |
| Infections and infestations | 10 | (4.7) | 6 | (2.8) | 19 | (6.6) | 9 | (3.1) | 3 | (3.6) | 2 | (2.4) | 2 | (2.2) | 1 | (1.1) | 2 | (2.9) | 0 |  |
| Neoplasms benign, malignant, and unspecified (including cysts and polyps) | 1 | (0.5) | 1 | (0.5) | 1 | (0.3) | 1 | (0.3) | 1 | (1.2) | 1 | (1.2) | 0 |  | 0 |  | 0 |  | 0 |  |
| Blood and lymphatic system disorders | 7 | (3.3) | 3 | (1.4) | 4 | (1.4) | 3 | (1.0) | 0 |  | 0 |  | 2 | (2.2) | 1 | (1.1) | 0 |  | 0 |  |
| Immune system disorders | 0 |  | 0 |  | 0 |  | 0 |  | 0 |  | 0 |  | 0 |  | 0 |  | 0 |  | 0 |  |
| Endocrine disorders | 33 | (15.4) | 2 | (0.9) | 43 | (14.8) | 10 | (3.4) | 12 | (14.3) | 0 |  | 6 | (6.7) | 1 | (1.1) | 4 | (5.9) | 0 |  |
| Metabolism and nutrition disorders | 13 | (6.1) | 6 | (2.8) | 7 | (2.4) | 2 | (0.7) | 5 | (6.0) | 3 | (3.6) | 4 | (4.5) | 1 | (1.1) | 1 | (1.5) | 0 |  |
| Psychiatric disorders | 2 | (0.9) | 1 | (0.5) | 0 |  | 0 |  | 0 |  | 0 |  | 1 | (1.1) | 0 |  | 1 | (1.5) | 0 |  |
| Nervous system disorders | 7 | (3.3) | 1 | (0.5) | 3 | (1.0) | 0 |  | 1 | (1.2) | 1 | (1.2) | 3 | (3.4) | 3 | (3.4) | 2 | (2.9) | 2 | (2.9) |
| Eye disorders | 6 | (2.8) | 1 | (0.5) | 2 | (0.7) | 0 |  | 1 | (1.2) | 0 |  | 0 |  | 0 |  | 0 |  | 0 |  |
| Ear and labyrinth disorders | 0 |  | 0 |  | 0 |  | 0 |  | 0 |  | 0 |  | 0 |  | 0 |  | 0 |  | 0 |  |
| Cardiac disorders | 1 | (0.5) | 0 |  | 3 | (1.0) | 2 | (0.7) | 0 |  | 0 |  | 0 |  | 0 |  | 1 | (1.5) | 0 |  |
| Vascular disorders | 4 | (1.9) | 0 |  | 3 | (1.0) | 1 | (0.3) | 0 |  | 0 |  | 0 |  | 0 |  | 2 | (2.9) | 1 | (1.5) |
| Respiratory, thoracic and mediastinal disorders | 29 | (13.6) | 12 | (5.6) | 72 | (24.8) | 34 | (11.7) | 6 | (7.1) | 4 | (4.8) | 9 | (10.1) | 2 | (2.2) | 5 | (7.4) | 2 | (2.9) |
| Gastrointestinal disorders | 14 | (6.5) | 2 | (0.9) | 24 | (8.3) | 5 | (1.7) | 14 | (16.7) | 3 | (3.6) | 6 | (6.7) | 2 | (2.2) | 3 | (4.4) | 0 |  |
| Hepatobiliary disorders | 12 | (5.6) | 4 | (1.9) | 15 | (5.2) | 8 | (2.8) | 1 | (1.2) | 1 | (1.2) | 9 | (10.1) | 4 | (4.5) | 4 | (5.9) | 1 | (1.5) |
| Skin and subcutaneous tissue disorders | 27 | (12.6) | 4 | (1.9) | 22 | (7.6) | 1 | (0.3) | 10 | (11.9) | 0 |  | 10 | (11.2) | 2 | (2.2) | 9 | (13.2) | 1 | (1.5) |
| Musculoskeletal and connective tissue disorders | 14 | (6.5) | 2 | (0.9) | 8 | (2.8) | 2 | (0.7) | 4 | (4.8) | 0 |  | 2 | (2.2) | 1 | (1.1) | 2 | (2.9) | 1 | (1.5) |
| Renal and urinary disorders | 6 | (2.8) | 3 | (1.4) | 6 | (2.1) | 2 | (0.7) | 0 |  | 0 |  | 3 | (3.4) | 1 | (1.1) | 0 |  | 0 |  |
| Reproductive and breast disorders | 0 |  | 0 |  | 1 | (0.3) | 0 |  | 0 |  | 0 |  | 0 |  | 0 |  | 0 |  | 0 |  |
| Congenital, familial and genetic disorders | 0 |  | 0 |  | 0 |  | 0 |  | 0 |  | 0 |  | 0 |  | 0 |  | 0 |  | 0 |  |
| General and systemic disorders and administration site conditions | 15 | (7.0) | 0 |  | 21 | (7.2) | 4 | (1.4) | 7 | (8.3) | 1 | (1.2) | 9 | (10.1) | 1 | (1.1) | 3 | (4.4) | 1 | (1.5) |
| Investigations | 31 | (14.5) | 8 | (3.7) | 29 | (10.0) | 7 | (2.4) | 8 | (9.5) | 1 | (1.2) | 12 | (13.5) | 2 | (2.2) | 5 | (7.4) | 1 | (1.5) |
| Injury, poisoning and procedural complications | 5 | (2.3) | 0 |  | 18 | (6.2) | 1 | (0.3) | 5 | (6.0) | 0 |  | 1 | (1.1) | 0 |  | 4 | (5.9) | 0 |  |

Values are number (%) of patients

*ILD* interstitial lung disease, *SOC* system organ class, *TRAE* treatment-related adverse event

**ESM Table 4.** Frequency of TRAEs in patients with preexisting autoimmune diseases

| **Type of autoimmune disease** | **Patients** | **All TRAEs** | | | | **TRAESI** | | | |
| --- | --- | --- | --- | --- | --- | --- | --- | --- | --- |
|  |  | **Any grade** | | **Grade ≥3** | | **Any grade** | | **Grade ≥3** | |
|  | **N** | **n** | **(%)** | **n** | **(%)** | **n** | **(%)** | **n** | **(%)** |
| Rheumatoid arthritis | 41 | 24 | (58.5) | 10 | (24.4) | 14 | (34.1) | 8 | (19.5) |
| Autoimmune thyroiditis | 31 | 24 | (77.4) | 7 | (22.6) | 21 | (67.7) | 4 | (12.9) |
| Basedow’s disease | 22 | 12 | (54.5) | 4 | (18.2) | 11 | (50.0) | 3 | (13.6) |
| Psoriasis | 17 | 11 | (64.7) | 4 | (23.5) | 8 | (47.1) | 1 | (5.9) |
| Chronic gastritis | 11 | 7 | (63.6) | 5 | (45.5) | 5 | (45.5) | 2 | (18.2) |
| Colitis ulcerative | 9 | 6 | (66.7) | 2 | (22.2) | 4 | (44.4) | 1 | (11.1) |
| Polymyalgia rheumatica | 9 | 2 | (22.2) | 0 |  | 2 | (22.2) | 0 |  |
| Type 1 diabetes mellitus | 7 | 6 | (85.7) | 2 | (28.6) | 5 | (71.4) | 2 | (28.6) |
| Sarcoidosis | 7 | 5 | (71.4) | 0 |  | 3 | (42.9) | 0 |  |
| Primary biliary cholangitis | 6 | 6 | (100.0) | 2 | (33.3) | 5 | (83.3) | 1 | (16.7) |
| Sjogren’s syndrome | 5 | 3 | (60.0) | 2 | (40.0) | 3 | (60.0) | 2 | (40.0) |
| Myasthenia gravis | 5 | 3 | (60.0) | 1 | (20.0) | 2 | (40.0) | 1 | (20.0) |
| Immune thrombocytopenia | 5 | 2 | (40.0) | 1 | (20.0) | 2 | (40.0) | 1 | (20.0) |
| IgA nephropathy | 5 | 2 | (40.0) | 0 |  | 2 | (40.0) | 0 |  |
| Rheumatic disorder | 4 | 3 | (75.0) | 1 | (25.0) | 1 | (25.0) | 1 | (25.0) |
| Autoimmune hepatitis | 4 | 2 | (50.0) | 1 | (25.0) | 2 | (50.0) | 1 | (25.0) |
| Systemic lupus erythematosus | 3 | 2 | (66.7) | 1 | (33.3) | 2 | (66.7) | 1 | (33.3) |
| Still's disease | 3 | 1 | (33.3) | 0 |  | 1 | (33.3) | 0 |  |
| Guillain–Barre syndrome | 2 | 2 | (100.0) | 0 |  | 2 | (100.0) | 0 |  |
| Alopecia areata | 2 | 2 | (100.0) | 0 |  | 1 | (50.0) | 0 |  |
| Dermatomyositis | 2 | 1 | (50.0) | 0 |  | 0 |  | 0 |  |
| Behcet’s syndrome | 2 | 0 |  | 0 |  | 0 |  | 0 |  |
| Multiple sclerosis | 2 | 0 |  | 0 |  | 0 |  | 0 |  |
| Vogt–Koyanagi–Harada disease | 2 | 0 |  | 0 |  | 0 |  | 0 |  |
| Cardiac sarcoidosis | 1 | 1 | (100.0) | 1 | (100.0) | 1 | (100.0) | 1 | (100.0) |
| Cold type hemolytic anemia | 1 | 1 | (100.0) | 1 | (100.0) | 1 | (100.0) | 1 | (100.0) |
| Crohn’s disease | 1 | 1 | (100.0) | 1 | (100.0) | 1 | (100.0) | 0 |  |
| Collagen disorder | 1 | 1 | (100.0) | 0 |  | 1 | (100.0) | 0 |  |
| Henoch–Schonlein purpura | 1 | 1 | (100.0) | 0 |  | 1 | (100.00) | 0 |  |
| SAPHO syndrome | 1 | 1 | (100.0) | 0 |  | 1 | (100.0) | 0 |  |
| Thromboangiitis obliterans | 1 | 1 | (100.0) | 0 |  | 1 | (100.0) | 0 |  |
| Vitiligo | 1 | 1 | (100.0) | 0 |  | 1 | (100.0) | 0 |  |
| Autoimmune hemolytic anemia | 1 | 1 | (100.0) | 1 | (100.0) | 0 |  | 0 |  |
| Renal amyloidosis | 1 | 1 | (100.0) | 1 | (100.0) | 0 |  | 0 |  |
| Neuromyelitis optica spectrum disorder | 1 | 1 | (100.0) | 0 |  | 0 |  | 0 |  |
| Pernicious anemia | 1 | 1 | (100.0) | 0 |  | 0 |  | 0 |  |
| Alveolar proteinosis | 1 | 0 |  | 0 |  | 0 |  | 0 |  |
| Cystitis interstitial | 1 | 0 |  | 0 |  | 0 |  | 0 |  |
| Gastrointestinal amyloidosis | 1 | 0 |  | 0 |  | 0 |  | 0 |  |
| Henoch–Schonlein purpura nephritis | 1 | 0 |  | 0 |  | 0 |  | 0 |  |
| Kawasaki's disease | 1 | 0 |  | 0 |  | 0 |  | 0 |  |
| Mixed connective tissue disease | 1 | 0 |  | 0 |  | 0 |  | 0 |  |
| Scleroderma | 1 | 0 |  | 0 |  | 0 |  | 0 |  |
| Vasculitis | 1 | 0 |  | 0 |  | 0 |  | 0 |  |

*SAPHO* synovitis-acne-pustulosis-hyperostosis-osteitis, *TRAE* treatment-related adverse event, *TRAESI* treatment-related adverse events of special interest

**ESM Table 5.** Incidence and recovery/remission of ILD in patients with or without a history of ILD

| **Category^a^** | **History of ILD**  **(N = 290)** | | | | | | | | **No history of ILD**  **(N =7131)** | | | | | | | |
| --- | --- | --- | --- | --- | --- | --- | --- | --- | --- | --- | --- | --- | --- | --- | --- | --- |
|  | **Any grade** | | **Grade 3** | | **Grade 4** | | **Grade 5** | | **Any grade** | | **Grade 3** | | **Grade 4** | | **Grade 5** | |
| Interstitial lung disease | 70 | (24.1) | 17 | (5.9) | 10 | (3.4) | 5 | (1.7) | 453 | (6.4) | 112 | (1.6) | 22 | (0.3) | 46 | (0.6) |
| Recovered/remission^b^ | 49 | (70.0) | 14 | (82.4) | 5 | (50.0) | - | - | 336 | (74.2) | 91 | (81.3) | 11 | (50.0) | - | - |

Values are number (%) of patients

*ILD* interstitial lung disease, *TRAESI* treatment-related adverse events of special interest

^a^Each category aggregates TRAEs with related preferred terms

^b^The denominator was the number of patients with ILD within each grade

**ESM Table 6.** TRAE SOCs in patients with a history of vaccination (N = 89)

| **SOC** | **Any grade** | | **Grade ≥3** | |
| --- | --- | --- | --- | --- |
| Any TRAEs | 56 | (62.9) | 10 | (11.2) |
| Infections and infestations | 7 | (7.9) | 3 | (3.4) |
| Neoplasms benign, malignant, and unspecified (including cysts and polyps) | 1 | (1.1) | 0 |  |
| Blood and lymphatic system disorders | 2 | (2.2) | 1 | (1.1) |
| Immune system disorders | 0 |  | 0 |  |
| Endocrine disorders | 15 | (16.9) | 0 |  |
| Metabolism and nutrition disorders | 8 | (9.0) | 3 | (3.4) |
| Psychiatric disorders | 0 |  | 0 |  |
| Nervous system disorders | 2 | (2.2) | 0 |  |
| Eye disorders | 0 |  | 0 |  |
| Ear and labyrinth disorders | 0 |  | 0 |  |
| Cardiac disorders | 1 | (1.1) | 0 |  |
| Vascular disorders | 0 |  | 0 |  |
| Respiratory, thoracic and mediastinal disorders | 6 | (6.7) | 0 |  |
| Gastrointestinal disorders | 10 | (11.2) | 1 | (1.1) |
| Hepatobiliary disorders | 6 | (6.7) | 1 | (1.1) |
| Skin and subcutaneous tissue disorders | 16 | (18.0) | 1 | (1.1) |
| Musculoskeletal and connective tissue disorders | 4 | (4.5) | 1 | (1.1) |
| Renal and urinary disorders | 2 | (2.2) | 1 | (1.1) |
| Reproductive and breast disorders | 0 |  | 0 |  |
| Congenital, familial and genetic disorders | 0 |  | 0 |  |
| General and systemic disorders and administration site conditions | 13 | (14.6) | 1 | (1.1) |
| Investigations | 9 | (10.1) | 0 |  |
| Injury, poisoning and procedural complications | 4 | (4.5) | 0 |  |

Values are number (%) of patients

*SOC* system organ class, *TRAE* treatment-related adverse event

**ESM Table 7.** TRAE SOCs in patients aged <75 or ≥75 years

| **SOC** | **Patients aged <75 years**  **(N = 5802)** | | | | **Patients aged ≥75 years**  **(N = 1619)** | | | |
| --- | --- | --- | --- | --- | --- | --- | --- | --- |
|  | **Any grade** | | **Grade ≥3** | | **Any grade** | | **Grade ≥3** | |
| Any TRAEs | 2806 | (48.4) | 960 | (16.5) | 837 | (51.7) | 277 | (17.1) |
| Infections and infestations | 192 | (3.3) | 93 | (1.6) | 69 | (4.3) | 36 | (2.2) |
| Neoplasms benign, malignant, and unspecified (including cysts and polyps) | 29 | (0.5) | 14 | (0.2) | 8 | (0.5) | 4 | (0.2) |
| Blood and lymphatic system disorders | 86 | (1.5) | 48 | (0.8) | 24 | (1.5) | 17 | (1.1) |
| Immune system disorders | 7 | (0.1) | 4 | (<0.1) | 1 | (<0.1) | 1 | (<0.1) |
| Endocrine disorders | 703 | (12.1) | 77 | (1.3) | 211 | (13.0) | 24 | (1.5) |
| Metabolism and nutrition disorders | 225 | (3.9) | 96 | (1.7) | 75 | (4.6) | 26 | (1.6) |
| Psychiatric disorders | 17 | (0.3) | 5 | (<0.1) | 5 | (0.3) | 2 | (0.1) |
| Nervous system disorders | 167 | (2.9) | 42 | (0.7) | 52 | (3.2) | 15 | (0.9) |
| Eye disorders | 49 | (0.8) | 9 | (0.2) | 11 | (0.7) | 1 | (<0.1) |
| Ear and labyrinth disorders | 6 | (0.1) | 0 |  | 2 | (0.1) | 0 |  |
| Cardiac disorders | 44 | (0.8) | 18 | (0.3) | 18 | (1.1) | 10 | (0.6) |
| Vascular disorders | 48 | (0.8) | 18 | (0.3) | 14 | (0.9) | 6 | (0.4) |
| Respiratory, thoracic and mediastinal disorders | 493 | (8.5) | 203 | (3.5) | 150 | (9.3) | 65 | (4.0) |
| Gastrointestinal disorders | 501 | (8.6) | 135 | (2.3) | 120 | (7.4) | 30 | (1.9) |
| Hepatobiliary disorders | 250 | (4.3) | 101 | (1.7) | 77 | (4.8) | 22 | (1.4) |
| Skin and subcutaneous tissue disorders | 582 | (10.0) | 67 | (1.2) | 185 | (11.4) | 20 | (1.2) |
| Musculoskeletal and connective tissue disorders | 120 | (2.1) | 20 | (0.3) | 53 | (3.3) | 17 | (1.1) |
| Renal and urinary disorders | 92 | (1.6) | 29 | (0.5) | 25 | (1.5) | 6 | (0.4) |
| Reproductive and breast disorders | 4 | (<0.1) | 0 |  | 2 | (0.1) | 0 |  |
| Congenital, familial and genetic disorders | 1 | (<0.1) | 1 | (<0.1) | 0 |  | 0 |  |
| General and systemic disorders and administration site conditions | 428 | (7.4) | 37 | (0.6) | 128 | (7.9) | 17 | (1.1) |
| Investigations | 668 | (11.5) | 156 | (2.7) | 182 | (11.2) | 32 | (2.0) |
| Injury, poisoning and procedural complications | 176 | (3.0) | 20 | (0.3) | 45 | (2.8) | 6 | (0.4) |

Values are number (%) of patients

*SOC* system organ class, *TRAE* treatment-related adverse event

**ESM Figure 1.** Time to onset of TRAESI^a^


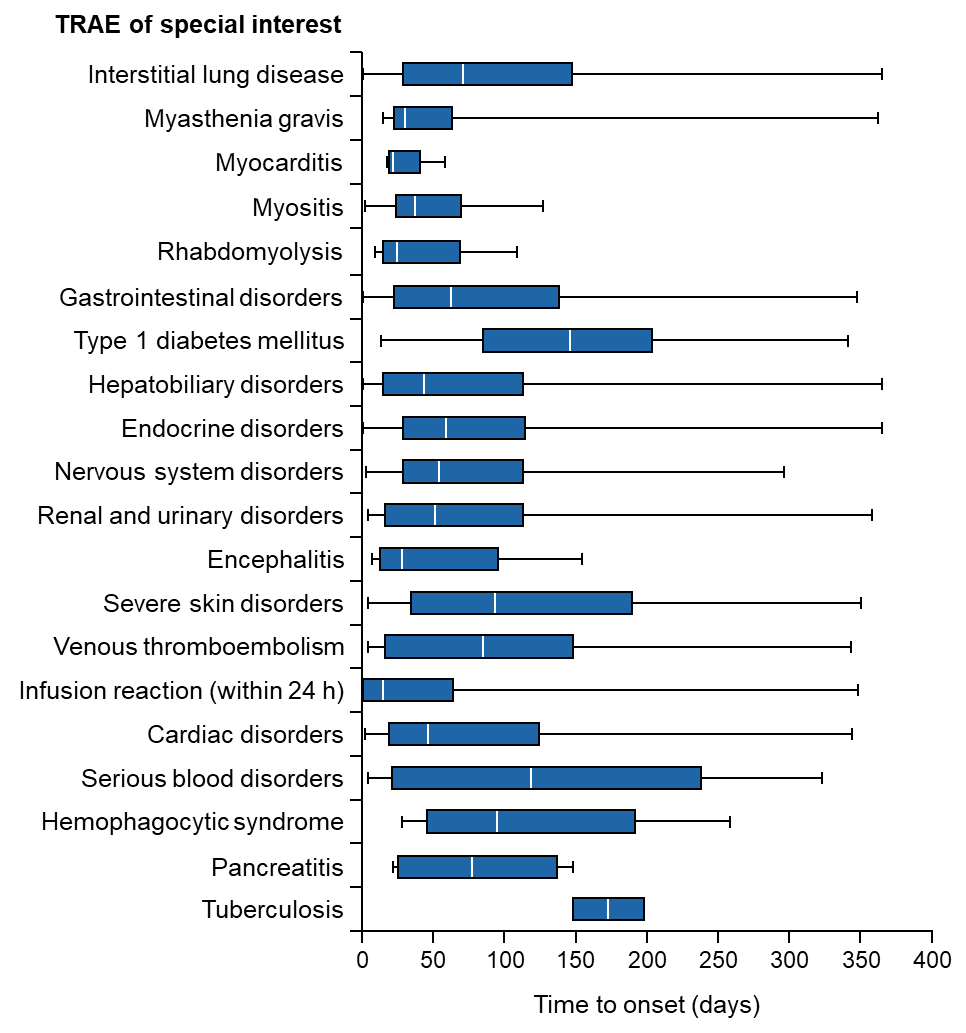


The white center line denotes the median value, the blue box shows the 25th to 75th percentiles, and the black whiskers mark the minimum (left) and maximum (right) values

*TRAESI* treatment-related adverse events of special interest

^a^Includes all adverse events related to the TRAESI
